# Accelerated enhanced recovery after colon cancer surgery with discharge within one day after surgery: a systematic review

**DOI:** 10.1186/s12885-023-11803-4

**Published:** 2024-01-18

**Authors:** Misha A. T. Sier, Anke H. C. Gielen, Thaís T. T. Tweed, Noémi C. van Nie, Tim Lubbers, Jan H. M. B. Stoot

**Affiliations:** 1 Department of Surgery, Zuyderland Medical Centre, Heerlen, the Netherlands; 2 Department of Surgery, Maastricht University Medical Centre+, Maastricht, the Netherlands; 3 School of Nutrition and Translational Research in Metabolism (NUTRIM), Maastricht University, Maastricht, The Netherlands; 4 GROW School for Oncology and Reproduction, Maastricht University, Maastricht, The Netherlands

**Keywords:** Colorectal surgery, Accelerated recovery, Enhanced recovery after surgery, Laparoscopic surgery

## Abstract

**Background:**

Recent studies have demonstrated that accelerated enhanced recovery after colorectal surgery is feasible for specific patient populations. The accelerated enhanced recovery protocols (ERP) tend to vary, and the majority of studies included a small study population. This hampers defining the optimal protocol and establishing the potential benefits. This systematic review aimed to determine the effect of accelerated ERPs with intended discharge within one day after surgery.

**Methods:**

PubMed (MEDLINE), Embase, Cochrane and Web of Science databases were searched using the following search terms: colon cancer, colon surgery, accelerated recovery, fast track recovery, enhanced recovery after surgery*.* Clinical trials published between January 2005 – February 2023, written in English or Dutch comparing accelerated ERPs to Enhanced Recovery After Surgery (ERAS) care for adult patients undergoing elective laparoscopic or robotic surgery for colon cancer were eligible for inclusion.

**Results:**

Thirteen studies, including one RCT were included. Accelerated ERPs after colorectal surgery was possible as LOS was shorter; 14 h to 3.4 days, and complication rate varied from 0–35.7% and readmission rate was 0–17% in the accelerated ERP groups. Risk of bias was serious or critical in most of the included studies.

**Conclusions:**

Accelerated ERPs may not yet be considered the new standard of care as the current data is heterogenous, and data on important outcome measures is scarce. Nonetheless, the decreased LOS suggests that accelerated recovery is possible for selected patients. In addition, the complication and readmission rates were comparable to ERAS care, suggesting that accelerated recovery could be safe.

**Supplementary Information:**

The online version contains supplementary material available at 10.1186/s12885-023-11803-4.

## Background

Since the introduction of the Enhanced Recovery After Surgery (ERAS) protocol by Kehlet et al. [1], there is increasing interest in recovery after surgery. The evidence-based interventions of the ERAS protocol have shown to reduce perioperative stress, maintain postoperative physiological functioning and accelerate recovery after surgery [2]. When combined with laparoscopic surgery, ERAS care effectuated a reduced Length Of hospital Stay (LOS) and morbidity, faster recovery with no increase in readmissions, and cost reduction compared with traditional care [1, 3–8].

The positive effects of ERAS piqued clinicians' interest in the possibility of further accelerating recovery by improving perioperative care. Recent studies have shown that an accelerated enhanced recovery protocol (ERP) is feasible for specific patients without compromising patients’ safety with discharge of all patients on the day of surgery to three days after colorectal surgery [9–16]. These results were obtained from studies that have explored ERPs with an optimized preoperative, perioperative, and postoperative protocol. The patient selection and accelerated ERP protocol differed between studies and included adjusted preoperative analgesia, Transversus Abdominis Plane (TAP) block during surgery, modified postoperative care with home visits by a domiciliary nurse or monitoring using a smartphone application [11, 12, 17]. The accelerated ERPs could potentially result in adaptation of perioperative care guidelines for colorectal cancer surgery. However, based on the current data, the optimal patient selection and protocol of an accelerated ERP is difficult to determine. Moreover, there is a certain ambiguity on what benefits this protocol could offer and which patients are suitable for accelerated recovery.

The aim of this systematic review was to determine the effect of accelerated ERPs with intended hospital discharge within one day after surgery compared to standard ERAS care on LOS, surgical outcomes, quality of life (QoL) and cost-effectiveness for patients undergoing elective surgery for colon cancer.

## Methods

This systematic review was prospectively registered in the PROSPERO database (CRD42023406341). The guidelines described in the Cochrane Handbook for Systematic Reviews of Interventions, Version 6.3, 2022 for the conduct of the review and the Preferred Reporting Items for Systematic Reviews and Meta-analysis of Individual Participant Data (PRISMA) guidelines for preparing the manuscript were followed [18, 19].

### Literature search

A systematic literature search in four key healthcare databases was conducted. PubMed (MEDLINE), Embase, Cochrane and Web of Science databases were searched from inception until February 10th, 2023. The following search terms were used: colon cancer, colon surgery, accelerated recovery, fast track recovery, enhanced recovery after surgery. For the literature search, there was no restriction on publication type or date. In addition, we searched trial registries (PROSPERO and ClinicalTrials.gov) for unpublished trials. Also, the reference lists of key records were assessed for additional relevant studies.

### Eligibility criteria

Clinical trials published from the first of January 2005, written in English or Dutch comparing an accelerated ERP to ERAS care for patients undergoing elective surgery for colon cancer were eligible for inclusion. This time restriction was selected since the first ERAS guideline was published in 2005, therefore it would be very unlikely that any relevant trials would be found prior to this year [1]. Studies were not restricted on study type but had to include at least a study population of 10 patients, with an age ≥ 18 years. To create a homogenous patient population, studies had to involve patients undergoing elective laparoscopic or robot-assisted surgical resections for colon cancer. Studies that included only benign or rectal surgery were excluded,

### Study selection

The results of the literature search were collected and de-duplicated in the Rayyan software [20]. All articles were first screened for relevance on title and abstract independently by two reviewers MS and AG. Full text articles were retained if they met the eligibility criteria described above. After retrieving and examining the full text of all potentially relevant articles, both reviewers indicated independently if the study should be included. Any discrepancies were double-checked and resolved by discussion with other members of the review team (JS and TL). When there were multiple reports featuring the same dataset, the publication with the longest duration of follow-up was included to diminish overlap and to include the largest patient population. If the study selection would yield less than 500 patients included in the accelerated ERP, also non-comparative ERP studies would be described.

### ERPs

We defined accelerated ERPs as accelerated enhanced recovery protocols with intended discharge within one day after surgery. Same day discharge (SDD) protocols were defined as protocols with intended discharge < 24 h after surgery without overnight hospital stay. Protocols that included overnight stay, were classified as ≤ 24 h stay protocols. Given the variation in protocol adaptations of ERPs, no restrictions on ERP elements were defined. Since the implementation of ERAS protocols varies between hospitals, we have set the limit on at least *7* ERAS items used in the control (ERAS) group to create a consistency in the provided ERAS care. This is in line with the Cochrane review of Spanjersberg et al. [3].

### Outcomes

The following data were extracted systematically from the included papers: author, publication year, study design, type of ERP and ERAS protocol, number of participants, patients’ characteristics, surgical data, i.e., indication for surgery and type of resection, and clinical outcomes. The primary outcome of this study was primary LOS (defined as number of days admitted after surgery). Secondary outcomes were total LOS (defined as number of days admitted after surgery and potential readmission days within 90 days after surgery), overall complications within 90 days after surgery (graded by the Clavien Dindo classification [21]), readmissions within 90 days after surgery, Quality of Life (QoL) and cost-effectiveness. If available, QoL was determined using disease specific PROMs [22]. Costs included only intramural hospital costs and were reported in the monetary unit of the study.

### Assessment of risk of bias in included studies

The potential source of bias of the included randomized trials was assessed using the RoB 2-tool [23], for non-randomized studies the ROBIN-I tool was used [24]. The robvis-tool was used to create risk-of-bias plots [25]. Each study was assessed using this method by reviewers MS and AG independently. Discrepancies were solved by consensus discussion with a third reviewer JS, if necessary.

The outcomes of all studies were reported using the Grading of Recommendation, Assessment, Development and Evaluation (GRADE) system [18].

### Statistical analysis

We extracted all data use from the original studies. To quantify the statistical heterogeneity in the studies, the I^2^ value was used. Only if studies were sufficiently clinically, methodologically, and statistically homogenous, the data were pooled in a meta-analysis. In case of considerable heterogeneity (> 75%), descriptive quantitative analysis and qualitative analysis would be performed and outcomes between included studies described [18].

## Results

### Selected studies

**Fig. 1:**
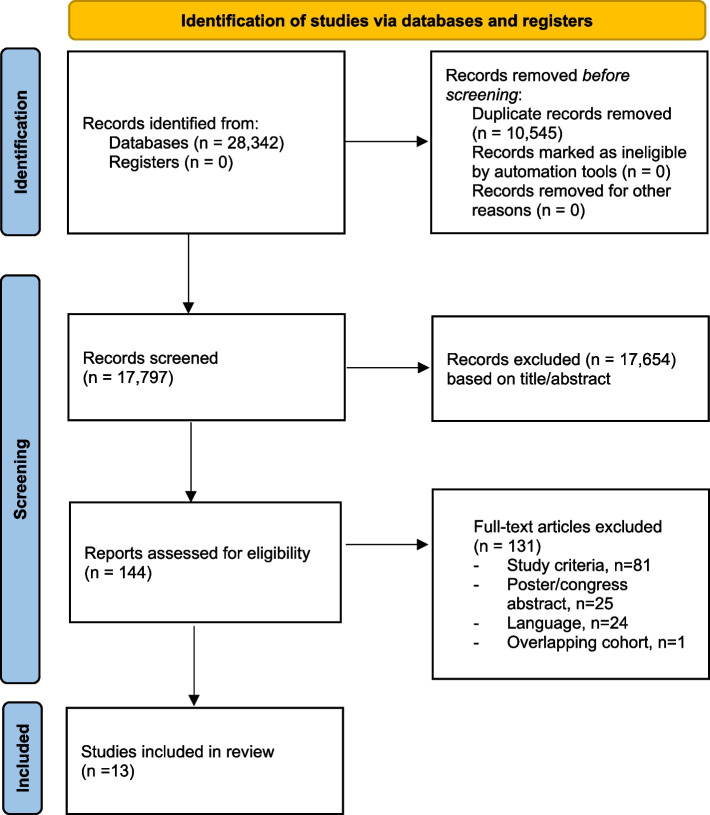
PRISMA flow diagram of the literature search and selection procedure

This systematic search identified 28,342 articles. All references from the four search databases were imported into Rayyan bibliographic software. The literature search and selection processes are shown in Fig. 1. After removing the duplicates, a total of 17,797 references were screened and assessed for eligibility based on title and abstract. Of these, 144 full-text articles were assessed for eligibility of which 5 studies met the inclusion criteria. Due to the limited number of included patients, non-comparative, and retrospective studies describing accelerated ERPs were also included. No additional studies were included based on reference check. In total 13 studies were included [9, 11, 12, 17, 26–34]. The baseline characteristics of the included studies, patient demographics and outcomes are shown in Tables 1 and 2.

**Table 1:** Characteristics included comparative studies, patient demographics, ERP versus ERAS, and outcomes

Author/year	Nr patients (I^1^/C^2^)	Intervention	Control	Primary LOS (I^1^/C^2^)	Total LOS (^1^/C^2^)	Readmissions (^1^/C^2^)	Complications (^1^/C^2^)	Reoperation (^1^/C^2^), n (%)	Mortality (^1^/C^2^)	Other
Bednarski et al. [32]; 2019	14/16	MIS^3^, ERAS and telemonitoring	ERAS	27.1/ 51.5 (median; hr)	28.3/51.5 (median; hr)	14.3/0%	35.7/0%	2/0 (14.3/0)	0/0 (30 days)	I^1^: CD^4^ ≥ III: 2, CD^4^ I-II: 3 C^2^: -
Kiran et al. [28]; 2022	87/88	< 24 h ERP	ERP	29 pts (33.3%) discharged < 24 h, mean LOS 17.2–109 / 82.3 h	NI^8^	9.2/8%	24.1/22.7%	2/0 (3.4/0)	0/NI^8^	I^1^: 9 blood transfusions, 5 ileus, 3 AL^5^, 2 SSI^6^ C^2^: 9 blood transfusions, 5 ileus, 4 SSI^6^, 1 AL^5^
Lee et al. [12]; 2022	48/73	SDD^7^ ERP	ERP	0/2 (median; days)	NI^8^	6/4%	17/15%	NI^8^	NI^8^	I^1^: 2 SSI^6^, 2 genitourinary compl., 2 bleeding, 2 other, 1 AL^5,^ 1 cardiopulmonary compl. C^2^: 4 ileus, 3 AL^5^, 2 cardiopulmonary compl. 2 genitourinary compl., 1 bleeding, 1 other
Popeskou et al. [33]; 2022	51/782	ERAS with discharge < 24 h protocol	ERAS	6% (51) discharged < 24 h	NI^8^	7.8/9.2%	NI^8^	3.9/2.9%	0/2 (0/0.3%)	I^1^: 47 of 51 patients had uneventful recoveries
Tweed et al. [26]; 2022	41/74	23 h ERP	ERAS	80% discharge < 24 h	NI^8^	17.1/5.3%	31.7/26.7%	4.9/8.0%	0/0	I^1^: 4 bleeding, 4 bladder ret., 2 ileus, 2 infections, 1 AL^5^, 1 electrolyte deficiency C^2^: 6 infections, 4 AL^5^, 2 abscess, 2 anemia, 2 AF^9^, 2 electrolyte deficiency, 1 bowel ischemia, 1 bladder ret., 1 ileus, 1 tachycardia, 1 embolus

^1^*I* Intervention (ERP)

^2^*C* Control (ERAS)

^3^*MIS* Minimally Invasive Surgery

^4^*CD* Clavien Dindo

^5^*AL* anastomotic leakage

^6^*SSI* surgical site infection

^7^*SDD* Same-Day Discharge

^8^*NI* not investigated

^9^*AF* atrial fibrillation

**Table 2:** Characteristics included non-comparative studies, patient demographics, ERP versus ERAS, and outcomes

Author/year	Nr patients	Intervention	Primary LOS	Total LOS	Readmissions	Complications	Reoperation	Mortality	Other
de Azevedo et al. [34]; 2021	664	< 24 h ERP	237 pt discharged < 24 h (35.7%)	NI^1^	16 (6.8%) of patients discharged < 24 h	NI^1^	2 of patients discharged < 24 h (0.8%)	No morality among early discharged patients	Causes readmission early discharged patients: 10 ileus, 3 AL^2^, 1 SSI^3^, 1 internal hernia, 1 incisional hernia
Chasserant et al. [30]; 2016	40	Outpatient colectomy protocol	97.5% SDD^4^ (39 pt)	NI^1^	0	2.5%	0	0%	NI^1^
Curfman et al. [27]; 2022	115	SDD^4^ protocol	100% SDD^4^	NI^1^	0.9%	NI^1^	0	0	NI^1^
Favuzza et al. [31]; 2013	100	ERP with TAP^5^ block	27% discharge POD1, 62% discharged < 48 h, median LOS 2 days	NI^1^	2%	8%	NI^1^	0%	NI^1^
Lee et al. [17]; 2022	105 (site 1: 70/site 2: 35)	SDD^4^ ERP	Success rate SDD^3^: 80/63%, LOS 1 (both sites, median; days)	NI^1^	9/14%	16/20%	NI^1^	NI^1^	4 genitourinary compl., 4 other, 3 bleeding, 3 SSI^3^, 2 ileus, 2 AL^2^, 2 infections, 1 cardiopulmonary compl.
Levy et al. [9]; 2009	10	23 h ERP	0.95 (median; days), 100% discharged < 24 h	0.95 (median; days)	0	0	0	0%	-
Seux et al. [11]; 2022	177	Ambulatory ERP	14 (mean; hours)	NI^1^	9%	14.7%	8 (4.5%)	0%	7 AL^2^ fistula, 4 anastomotic haemorrhage, 1 ileocolic bleeding
Studniarek et al. [29]; 2020	360	ERP with one night hospital stay	21.7% discharged < 24 h, LOS 3.4 (mean; days)	NI^1^	8.6%	13.6%	1 (0.3%)	0.3%	CD^6^ I: 30 pt, CD^6^ II: 9, CD^6^ IIIa: 2, CD^6^ V: 1

^1^*NI* Not investigated

^2^*AL* Anastomotic Leakage

^3^*SSI* surgical site infection

^4^*SDD* Same-Day Discharge (discharge on day of surgery)

^5^*TAP* Transversus Abdominis Plane

^6^*CD* Clavien Dindo classification

### Study characteristics

Of the studies included, five studies were prospective comparative cohort studies [12, 26, 28, 32, 33]. The non-comparative studies included five prospective cohort studies [9, 11, 17, 30, 31] and three were retrospective cohort studies [27, 29, 34]. Of these thirteen included studies, six studied a same-day discharge (SDD) protocol [11, 12, 17, 27, 29, 30] and seven studied a ≤ 24-h stay pathway [9, 26, 28, 31–34]. The total sample size of these 13 studies consisted of 2,798 patients undergoing colorectal surgery, 1,337 patients were treated according to an accelerated ERP compared with 1,461 patients receiving standard care.

### Risk of bias

**Fig. 2:**
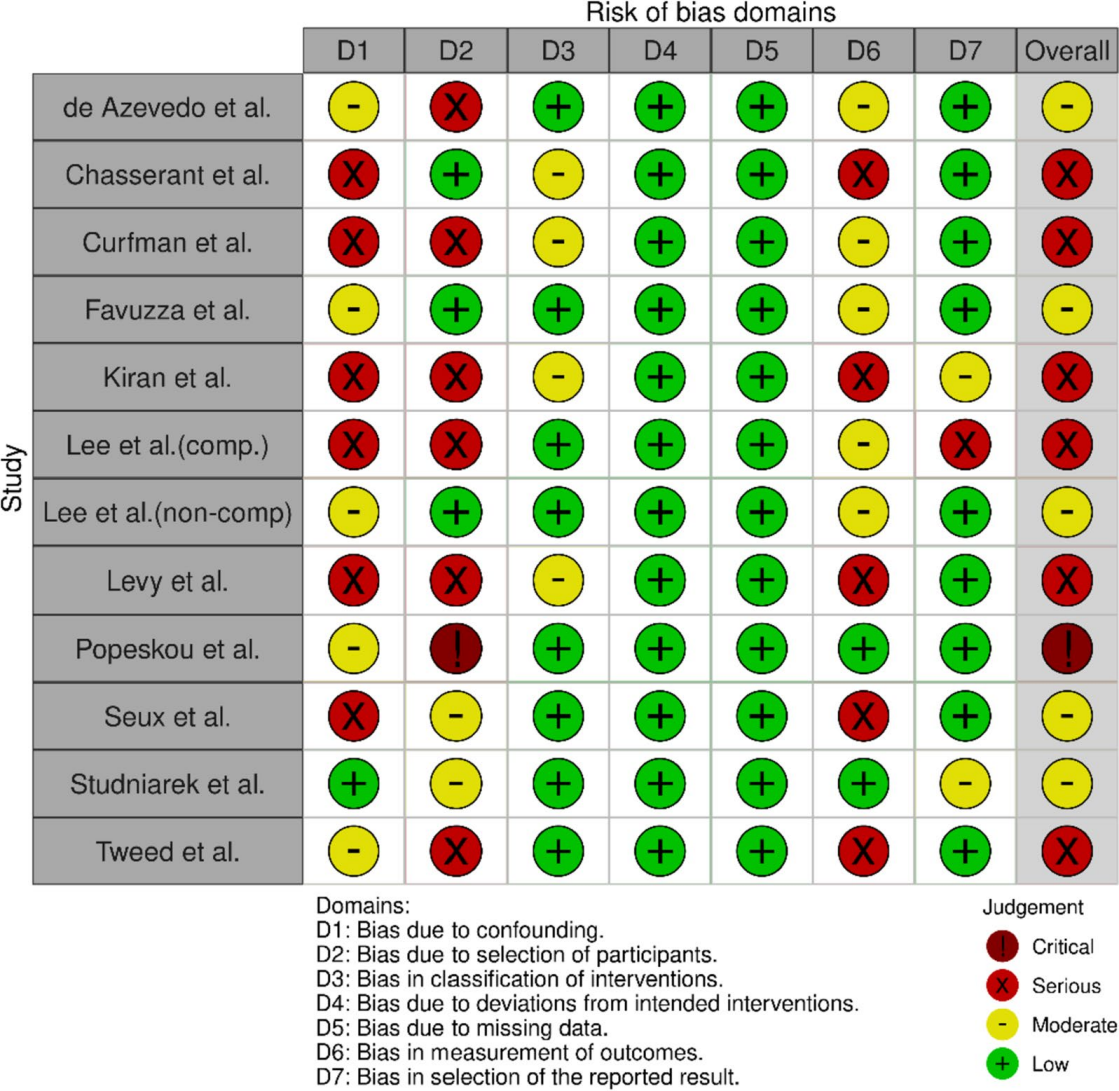
ROBINS-I plot risk of bias

**Fig. 3:**
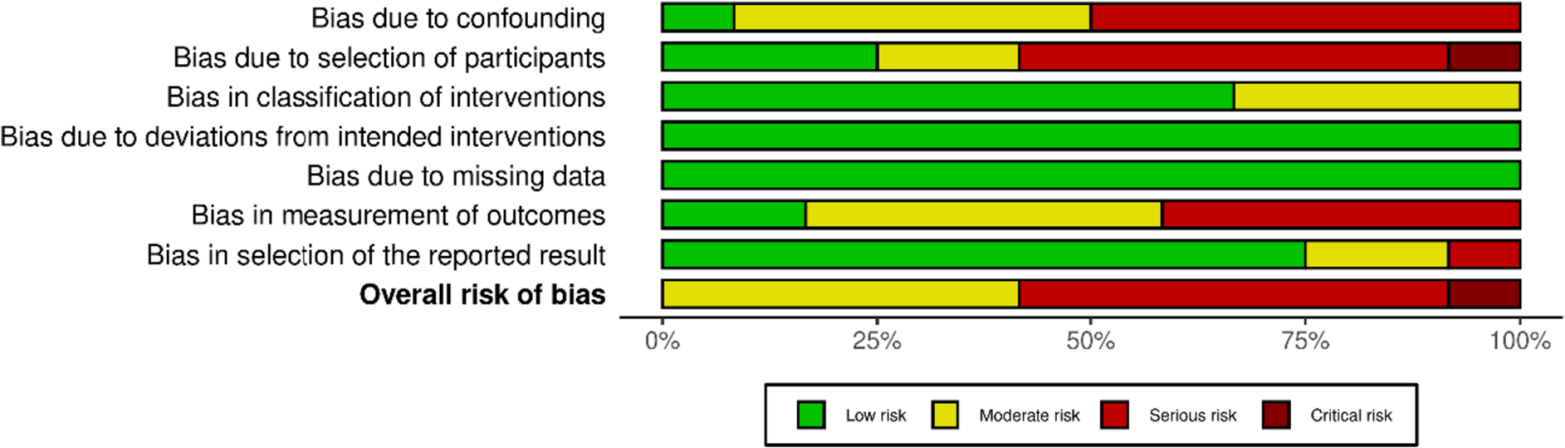
ROBINS-I weighted summary plot

**Fig. 4:**
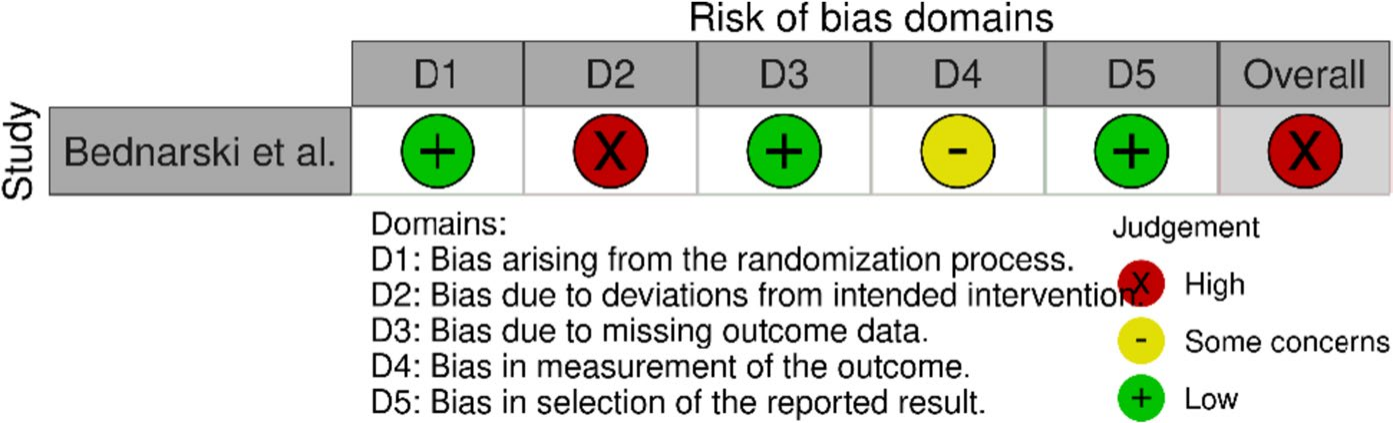
ROBINS-II plot risk of bias

**Fig. 5:**
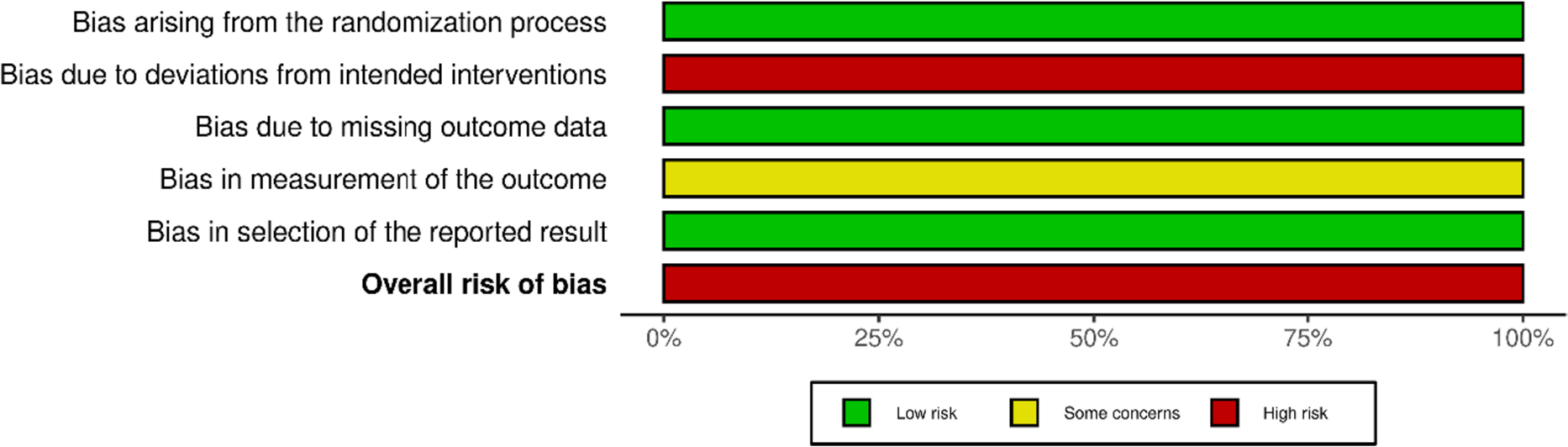
Cochrane RoB2 graph: Review authors' judgements about each risk of bias item presented as percentages across included randomized study

The methodological quality is presented in risk of bias (RoB) summaries (Figs. 2, 3, 4, 5). Blinding of participants and personnel was not possible due to the nature of the intervention. Eight studies had a serious or critical risk of bias [9, 12, 26–28, 30, 32, 33], which was due to confounding, selection bias, bias in measurement or bias in the selection of reported results. Five cohort studies had moderate risk of bias [11, 17, 29, 31, 34]. Overall, the methodological quality of the included studies ranged from moderate to low. Due to the heterogeneity of studies, GRADE criteria could not be applied.

### ERP protocol

Upon analysing the ERP groups, studies applied different modifications to or put extra emphasis on specific ERAS elements during the pre-operative, peri- and postoperative phases. An overview of the pre-, peri-, postoperative and post discharge protocol adjustments of the accelerated ERPs is listed in Table 3.

**Table 3:** Overview of specific ERAS targets in accelerated ERPs

Author/year	Pre-operative	Perioperative	Post-operative	After discharge
de Azevedo et al. [34]; 2021	-No mechanical bowel preparation -Oral AB^1^ -Admission at 07.00 AM	-Intraabdominal pressure 12 mmHg -Hyperoxygenation -Limited i.v. fluids -Local anaesthesia or TAP^2^ block	-Prokinetics -Regular analgesics	
Bednarski et al. [32]; 2019	-Mechanical bowel preparation & oral AB^1^ -Adjusted analgesia -GD^3^ fluid management	-I.v. dexamethasone -Narcotic sparing anaesthesia -Fluid optimization -MIS^4^	-Early intake -Discharge POD^5^ 1	-Teleconsulting POD^5^ 2 -Outpatient i.v. fluid hydration if necessary
Chasserant et al. [30]; 2016	-Counselling -Dietary intervention (low residual diet) -Colon preparation	-TCIVA^6^ -Adjusted analgesia including TAP^2^ block, nefopam -MIS^4^	-Chewing gum -Early mobilization and intake -Discharge POD^5^ 0	-Surveillance at home by visiting nurse POD^5^ 0–4 2x/day, POD^5^ 5–10 daily -Daily transmission clinical data -Daily phone call surgical assistant -Lab test POD^5^ 1, 3, 5
Curfman et al. [27]; 2022		-MIS^4^	-Discharge from PACU^7^ POD0 -Adjusted anti-emetics and analgesia	-Telephone consult POD^5^ 1&3 -Visit outpatient clinic POD^5^ 5&7
Favuzza et al. [31]; 2013		-MIS^4^ -TAP^2^ block	-Early mobilization and intake -Spirometry hourly -Discharge POD^5^1	-Telephone consult < 48 h after surgery
Kiran et al. [28]; 2022	-Counselling -Adjusted analgesia -Mechanical bowel preparation& oral AB^1^ -Antiseptic shower	-Specimen extraction with wound protector -Wound infiltration	-Adjusted anti-emetics and analgesia -Lab at PACU^6^ -Discharge from PACU^6^ POD^5^ 0	-Telephone consult POD^5^ 1
Lee et al. [17]; 2022	-Download mobile phone application (site 1) -First operation of the day	-MIS^4^ -TAP^2^ block	-Monitoring at PACU^6^ 4-6 h -Opioid-sparing analgesics -Early mobilization and intake	-One of the two sites offered daily health checks with a mobile app until POD^5^ 7
Lee et al. [12]; 2022	-Download mobile phone application -First operation of the day	-MIS^4^ -TAP^2^ block	-Monitoring at PACU^6^ 4-6 h -Opioid-sparing analgesics -Early mobilization and intake	-Daily health checks with a mobile app until POD^5^ 7
Levy et al. [9]; 2009	-Counselling -Adjusted analgesia -Avoidance bowel preparation -Surgery scheduled 2nd of the day	-Spinal anaesthesia -Oesophageal doppler for GD^3^ fluid therapy	-Analgesia - Early mobilization and intake -Discharge < 24 h	-Telephone consult evening of discharge (POD^5^ 1)
Popeskou et al. [33]; 2022	-Counselling	-Spinal anaesthesia -MIS^4^ -Wound infiltration	-Early mobilization and intake -Discharge < 24 h	-Daily telephone consult until POD^5^ 7
Seux et al. [11]; 2022	-Counselling -Immunonutrition 7 days -Admission surgery 7a.m	-Infiltration of ropivacaine at diaphragmatic domes -Laparoscopy with mini-laparotomy incisions -Opioid-sparing regimen	-No i.v. infusion -Oral analgesia only -Early mobilization and intake -Discharge 12 h after surgery	-Nutritional supplements -Chewing gum and oral magnesium -Clinical monitoring until POD^5^ 10 1x/day by nurse -Daily telephone consults until POD^5^ 5 -Lab check POD^5^ 2, 4 and 8
Studniarek et al. [29]; 2020	-Counselling	-Local wound infiltration	-Early discharge	-Mobile communication platform
Tweed et al. [26]; 2022	-Counselling -Adjusted analgesia -Mobile until surgery	-Spinal anaesthesia -Low intraabdominal pressure -MIS^4^ -Intracorporeal anastomosis -Restricted fluid infusion	-Analgesia -Early mobilization and intake -Discharge < 24 h	-Telephone consult POD^5^ 1 & 3

^1^*AB* Antibiotics

^2^*TAP* Transversus Abdominis Plane

^3^*GD* Goal-Directed

^4^*MIS* Minimally Invasive Surgery

^5^*POD* Post Operative Day

^6^*TCIVA* Target-Controlled Intravenous Anaesthesia

^7^*PACU* Post-Anesthesia Care Unit

### Heterogeneity

The studies varied clinically (e.g., patient population, type of ERP) and methodologically (e.g., cohort, randomized trial). Different outcome measures were reported in various ways across studies. Therefore, a meta-analysis was not feasible. A quantitative analysis was performed for the results of LOS, postoperative complications, readmissions, and patient satisfaction.

### Patients’ characteristics

Age, gender, ASA, indication for surgery (benign/malignant), and surgical procedures are displayed in Tables 4 and 5.

**Table 4:** Patients’ characteristics comparative studies

**Author**	**Age** (I^1^/C^2^) years	**Gender** M: F (I^1^/C^2^)	**ASA** (I^1^/C^2^)	**Indication for surgery** Benign: malignant (I^1^/C^2^)	**Types of surgery performed** (I^1^/C^2^)
Bednarski et al. [32]	58.7/59.3 (mean)	14: 16	II: 0 / 2 III: 14/ 14	1: 13 / 1: 15	Right colectomy: 8 / 8 Left colectomy: 3 / 1 LAR^3^: 3 / 7
Kiran et al. [28]	55.2–58.3/60.2 (mean)	40: 47 / 41: 47	I-II: 51 / 47 III: 36 / 41	Benign: 37 / 34 Malignancy/adenoma: 40 / 37 Other (not specified): 10 / 17	Ileocolic resection: 14 / 14 Right colectomy: 17 / 25 Transverse colectomy: 4 / 2 Left colectomy: 4 / 5 Sigmoid colectomy: 35 / 25 LAR^3^: 10 / 15 Subtotal colectomy: 3 / 4
Lee et al. [12]	60.2/56.5 (mean)	22: 26 / 43: 30	I: 4 / 4 II: 27 / 38 III: 17 / 31	19: 25 / 15: 47 Other (not specified): 3 / 3	Right colectomy: 14 / 33 Left/sigmoid colectomy: 12 / 22 LAR^3^: 7 / 11 Stoma closure: 15 / 7
Popeskou et al. [33]	67/70 (median)	34: 17 / 369: 413	I: 10 / 110 II: 35 / 482 III: 6 / 168 IV: 0 / 6	6: 45 / 192: 590	Ileocolonic resection: 1 / 156 Right hemicolectomy: 29 / 328 Extended right hemicolectomy: 0 / 63 Left hemicolectomy: 1 / 31 Sigmoid colectomy: 1 / 55 High anterior rectal resection: 19 / 282
Tweed et al. [26]	64.2/69.4 (mean*)*	27: 24 / 33: 42	I: 1 / 8 II: 40 / 67	0: 41 / 0: 75	Left hemicolectomy: 3 / 6 Right hemicolectomy: 19 / 28 High anterior resection: 18 / 37 Transverse colectomy: 1 / 1 Total colectomy: 0 / 1 Subtotal colectomy: 0 / 2

^1^*I* Intervention

^2^*C* Control

^3^*LAR* low anterior resection

**Table 5:** Patients’ characteristics non-comparative studies

**Author**	**Age** years	**Gender** M: F	**ASA**	**Indication for surgery** Benign: malignant	**Types of surgery performed**
de Azevedo et al. [34]	60 (mean)	255: 409	NI^1^	249: 414	Right colectomy: 130 Left colectomy: 26 Sigmoidectomy: 7 Partial colectomy: 28 Proctocolectomy: 8 Rectosigmoidectomy: 376 Abdominoperineal amputation: 18 Total colectomy 25 Other colorectal procedures: 46
Chasserant et al. [30]	56 (mean)	24: 16	I: 8 II: 24 III: 8	34: 6	Left colectomy: 33 Left colectomy combined with additional procedures^2^: 7
Curfman et al. [27]	NI^1^	48: 67	NI^1^	NI^1^	Cecectomy: 9 Right colectomy: 25 Transverse colectomy: 3 Left colectomy: 3 Sigmoidectomy: 6 LAR^3^: 61 Parastomal hernia: 5 Proctectomy: 3
Favuzza et al. [31]	60.5 (mean)	38: 62	NI^1^	Inflammatory: 31 Cancer or polyp: 65 Other^4^: 4	Right colectomy or ileocolic: 35 Left colectomy or sigmoid: 15 LAR^3^/Proctectomy/IPAA^5^: 34 Total colectomy: 4 Other^6^: 12
Lee et al. [17]	Site 1: 59.2 (mean) Site 2: 54.9 (mean)	29: 41 15: 20	NI^1^	Site 1 27: 33 Site 2 17: 17 Other (not specified): 6 (S1) / 1 (S2)	Right colectomy: 18 (S1) / 7 (S2) Left/sigmoid colectomy: 22 / 9 LAR^3^: 10 / 6 Stoma closure: 19 / 12 Small bowel resection: 1 / 1
Levy et al. [9]	60 (mean)	4: 6	I: 1 II: 9	1:9	Right colectomy: 3 Left colectomy: 1 Sigmoid colectomy: 2 High anterior resection: 2 Total mesorectal excisions: 2
Seux et al. [11]	65 (median)	94: 83	NI^1^	0: 177	Right colectomy: 72 Left colectomy: 89 Left angular or transverse colectomy: 16
Studniarek et al. [29]	64 (median)	161: 199	I: 8 II: 204 III: 139 IV: 8 V: 1	169: 191	Ileocecal resection: 15 Right hemicolectomy: 122 Transverse colectomy: 3 Left hemicolectomy: 17 Sigmoid colectomy: 1 Anterior resection: 110 LAR^3^: 48 Ultra-LAR^3^: 23 Subtotal colectomy: 12 Total colectomy: 9

^1^*NI* Not Investigated

^2^Additional resections: oophorectomy, salpingectomy, bladder dome resection, appendectomy, umbilical hernia repair, removal of umbilical mesh

^3^*LAR* Low Anterior Resection

^4^Other: Volvulus, recurrent obstruction or adhesions

^5^*IPAA* Ileal Pouch-Anal Anastomosis

^6^Other: Lysis of adhesions, ileostomy and colostomy

#### Primary outcome—Length of hospital stay

Nine studies described mean or median LOS, ranging from 14 h to 3.4 days [11, 29]. Success rate of SDD ranged from 63–97.5% [17, 30]. Discharge ≤ 24 h after surgery was achieved in 6–100% of the patient population [9, 33]. See Tables 1 and 2.

#### Secondary outcomes—Clinical outcomes

Most studies described complication rates, varying from 0 to 35.7% in comparative studies [9, 32] and 2.5–20% in non-comparative studies. The incidence of complications and readmission was higher in the intervention group in four of the five the studied ERP cohorts [12, 26, 28, 32]. The two studies that analysed difference in postoperative outcomes reported that this difference was not statistically significant [12, 26]. Three studies stated that the complications or reasons for readmission were unlikely to be related to the ERP [12, 26, 32]. For the accelerated discharge population, the complications included: ileus 0–7.1% [9, 27, 30, 32], anastomotic leakage 0–3.4% [9, 27, 28, 30]. The only study that reported a mortality rate higher than 0%, was the study of Studniarek et al. (0.3%, *n* = 1) [29].

#### Secondary outcomes—Patient satisfaction, QoL

Patients’ satisfaction with the ERP or QoL was reported in three studies; Bednarski et al. described no difference in patient satisfaction or QoL between groups, Tweed et al. and Lee et al. reported high patient satisfaction among the ERP patient population [12, 26, 32].

### In- and exclusion criteria/indications and contraindications

Some studies only included patients undergoing colon surgery [11, 26, 28, 33], while 8 studies also included patients with rectal surgery [12, 17, 27, 29, 30, 32, 34]. Both benign and malignant disease were included in 9 studies [9, 12, 17, 28–30, 32–34]. Nine studies included only elective laparoscopic or robotic surgical procedures [9, 11, 12, 17, 27, 28, 31–33]. Five studies only included patients living within proximity of the hospital [9, 12, 17, 28, 33]. Other inclusion criteria comprised patients owning a smart phone [12, 17], or being reachable by phone [26, 33], having no contraindications for a TAP block [12, 17, 31], having support at home by family or informal caregiver [11, 12, 17, 26, 33, 34].

Significant comorbidities were a reason for exclusion in 11 studies [9, 11, 12, 17, 26–28, 30, 32–34], as well as a history of laparotomy [30], major abdominal surgery [27] or severe nausea and vomiting [9, 32]. Patients were excluded in case of conversion from laparoscopic to open surgery [9, 27, 32], complex surgery [28, 29], multivisceral resection [12, 17], ostomy creation [9, 11, 12, 17, 26, 28, 32] or end colostomy [29], perioperative complication [9, 26, 27], total colectomy [27]. For some studies therapeutic anticoagulant therapy [30] or chronic opioid use were exclusion criteria [12, 17]. Seux et al. excluded patients living in an isolated or hostile environment [11].

### Discharge criteria

Patients had to meet the following criteria to be discharged; normal vital parameters [9, 26, 29, 31, 32, 34, 35], independent mobilization [12, 17, 26, 34], sufficient (fluid) intake [9, 29, 31, 32] without nausea or vomiting [12, 17, 26, 28, 34], flatus [26, 31, 33], voiding without urinary catheter [9, 12, 17, 26, 28], adequate control of pain [29, 31, 32, 34] with oral analgesics [12, 17, 26], no sign of bleeding [35] or other complications [12, 17], stable laboratory results [28], residence within 2–3 h from the hospital [32]. Two studies used a classification: a Modified PADSS score > 9 [30], Chung exit score [11].

### Cost-effectiveness/costs

None of the studies included provided data on cost-effectiveness of the ERP compared to standard care.

## Discussion

This systematic review evaluated the effect of accelerated ERPs with intended discharge within one day after colorectal surgery. Thirteen studies were included in this review, one RCT was identified. Due to heterogeneity, no meta-analysis could be performed. Risk of bias was serious or critical in the majority of included studies, level of evidence was considered low. The results of this systematic review demonstrate that accelerated ERPs after colorectal surgery are applicable for a selected patient population as LOS was shorter. The complication and readmission rates of the accelerated ERP group were similar to patients treated according to the current ERAS care. Upon implementing accelerated ERP, more robust research is needed to establish the optimal protocol and its effects.

Previous reviews have implied the potential of accelerated ERPs but stated that the body of evidence was relatively low due to heterogeneity of studies [36, 37]. New studies have been published recently [11, 17, 26, 28], therefore it was deemed important to conduct a new systematic review. A robust body of evidence in favour of accelerated ERP could reduce clinicians’ restraint to implement accelerated ERPs after colorectal surgery. Currently, there remains fear of increased readmissions, potential delays in treating complications and confusion due to the variety of adapted care elements.

A few studies reported primary LOS [9, 11, 12, 17, 29, 31, 32], which varied widely (14 h [11]– 3.4 days [29]). The LOS was remarkably lower among patients treated according to accelerated discharge protocol compared with the median LOS of 4 days of the entire Dutch patient population in as reported by the Dutch Colorectal Audit [38]. It is important to bear in mind that this major reduction in LOS could be attributed to the selection of a relatively healthy patient population, to the strict discharge criteria, to the approach and urgency of surgery or to the extensive follow-up. Therefore, this finding cannot be extrapolated to all patients. Nonetheless, these protocols may also reduce LOS for patient populations other than those studied. There is a potential bias from the fact that a primary goal of the accelerated ERP’s was to reduce LOS; this could have affected outcome parameter in comparative studies.

The readmission rate of this review was higher than described in previous reviews [36, 37]. The discrepancy could be partly explained by the larger number of patients included in this review [11, 17, 26, 28, 31, 32, 34]. Furthermore, the majority of the additionally included studies were prospective studies [11, 17, 26, 28, 31, 32], used broader inclusion criteria [28, 31] or had an older study population [26]. Two comparative studies reported a considerably higher readmission rate in the accelerated ERP population [26, 32]. The authors of both studies considered this difference in readmission rate to be unrelated to the intervention based on the reasons for readmission, being i.e. bowel obstruction due to a port-site hernia, rectal blood loss or pneumonia. Despite the higher readmission rate, the rate of serious complications was comparable [26]. As these complications were detected in a timely fashion, potential delay in diagnosis and treatment was low.

This review shows comparable readmission rates compared with the international readmission rate of 5.5–16% [39–41]. This supports the idea that accelerated ERPs do not increase readmission.

Accelerated recovery can only be considered to be successful if patient safety is guaranteed. The few studies that compared complication rates reported higher but not statistically significant differences in rates of minor complications in the group with accelerated recovery. The rates of anastomotic leakage were comparable between the two arms and mortality rates were low in both groups, suggesting that the accelerated ERP could be safe.

Even though the increase in minor complication will hardly affect patients’ safety or readmission rate, it could effectuate increase in emergency room visits or lower patient satisfaction. Few studies described emergency department (ED) visits [12, 17, 27, 29, 32], Two studies reported a slight increase in ED visits in the accelerated ERP group [12, 26, 32]. The complication rate of this review is higher than reported in previous reviews [36, 37]. The larger number of prospective studies could be a possible explanation for the higher rate of complications as well as differences in patient characteristics, indications for surgery and types of surgery [11, 17, 26, 30–32].

In general, complications occur in approximately 30% of patients after oncological colorectal resections [42, 43]. As this review includes mostly relatively healthy and young patients, it is to be expected that most reported complication rates in this review were lower than in the general surgical population. The generalizability of these results is limited since the way complications were reported across studies was not constant between studies, nor were definitions of complications or the complication rates. As method for follow-up was not defined in all studies, it remains unclear whether all complications were scored.

The included studies all used slightly different inclusion criteria, but most of the included subjects had a limited medical record. Thus, little data exists to guarantee safety of implementing an accelerated ERAS protocol in a population with extended co-morbidity or higher age.

A minority of the studies provided information about long term outcome parameters such as oncological survival or quality of life but available literature showed high patient satisfaction and quality of life [26, 32].The economic effects of the intervention have not been investigated yet. The number of studies performing cost-analysis is limited, but it is plausible that this reduction in LOS results in lower healthcare costs as long as it does not increase the use of healthcare after discharge.

### Strengths and limitations

This study has some limitations. First, the studies were heterogeneous in terms of the accelerated ERP protocols, length of follow-up and patient population, indications for surgery and types of surgical procedures. Several studies included patients undergoing colon and rectum cancer surgery, while these surgical procedures and the postoperative course differ [12, 17, 27, 29, 30, 32, 34, 38]. The difference between protocols may be due to the fact that exact knowledge on separate effects of modified or emphasized ERAS-elements is lacking. It has been shown that the strength of ERAS is the sum of its elements [3]. Nonetheless, due to the heterogeneity, studies are hardly comparable or applicable to a broader patient population. The ERAS protocol of the control cohort has not been described in detail; therefore, it is difficult to determine what ERAS-elements were incorporated into the standard care.

Available data provided little information regarding QoL, long-term outcomes, protocol adherence or implementation. Despite today's emphasis on reducing the rising cost of health care, none of the studies described cost-effectiveness. Some accelerated ERPs included extensive home monitoring; the financial impact would be worth noting. Overall risk of bias was moderate to critical. Only one RCT was included, all other studies were cohort studies. Methodological quality was low. Moreover, some studies included patients in the early discharge cohort based on postoperative measures. Therefore, the measured effects might be under- or overestimated. A strength of this review is the extensive literature search and updated overview of the current protocols and outcomes of accelerated ERPs. Further large clinical trial with a well-defined intervention and control group, which take clearly defined complications with a follow-up of at least 30 days, QoL, cost-effectiveness, protocol adherence, and its implementation into account, will need to be undertaken. These studies could initiate the development of a uniform accelerated ERP.

## Conclusion

Based on current evidence, this systematic review has shown, that the accelerated ERP may not yet be considered the new standard of care as the current data is heterogenous, and important outcome measures are lacking. However, since the results are promising in selected patients, there is need to conduct large, randomized trials with strict separation between accelerated ERP and ERAS protocols.

### Supplementary Information

**Additional file 1.**

## Data Availability

All data generated or analysed during this study are included in this published article and its supplementary information files.
