# Supplementary material for: Accelerated enhanced recovery after colon cancer surgery with discharge within one day after surgery: a systematic review
Source: BMC Cancer. 2024 Jan 18;24:102. doi: 10.1186/s12885-023-11803-4 (PMC10795207; doi:10.1186/s12885-023-11803-4)
Supplement: Supplementary file 1 — Additional file 1. [file 12885_2023_11803_MOESM1_ESM.docx]

**Pubmed**

| #17 | Search: **#15 AND #16** Sort by: **Most Recent** |
| --- | --- |
| #16 | Search: **#4 OR #5 OR #6 OR #7 OR #8 OR #9 OR #10 OR #11 OR #12 OR #13** Sort by: **Most Recent** |
| #15 | Search: **#1 OR #14** Sort by: **Most Recent** |
| #14 | Search: **#2 OR #3** Sort by: **Most Recent** |
| #13 | Search: **("day car*"[tiab] OR daycar*[tiab] OR "partial hospitali*"[tiab] OR "ambulatory car*"[tiab] OR outpatient*[tiab] OR "out patient*"[tiab] OR "office surg*"[tiab] OR "day surg*"[tiab] OR "ambulatory surg*"[tiab] OR "day of surgery discharg*"[tiab] OR "same day discharg*"[tiab] OR "ambulant surg*"[tiab] OR "ambulant therap*"[tiab] OR "ambulant car*"[tiab] OR "ambulatory treat*"[tiab] OR "ambulant treat*"[tiab] OR "outward patient*"[tiab] OR policlinic*[tiab] OR polyclinic*[tiab] OR "ambulatory colectom*"[tiab] OR "ambulant colectom*"[tiab] OR SDD[tiab] OR "same day colectom*"[tiab] OR "Outpatient monitoring"[tiab] OR "Ambulatory Monitoring"[tiab] OR "23 hour* of stay"[tiab] OR "23 hour* stay" [tiab] OR "23 hour* stays"[tiab] OR "24 hour* of stay"[tiab] OR "24 hour* stay" [tiab] OR "24 hour* stays"[tiab] OR "1 day* of stay"[tiab] OR "1 day* stay" [tiab] OR "one day* of stay"[tiab] OR OR "one day stay*"[tiab] OR "48 hour* of stay"[tiab] OR "48 hour* stay" [tiab] OR "48 hour* stays"[tiab] OR "2 day* of stay"[tiab] OR "2 day* stay" [tiab] OR "two day* of stay"[tiab] OR "two day* stay"[tiab] OR "two day* stays"[tiab] OR "one night stay*"[tiab] OR "one night of stay"[tiab] OR "1 night stay"[tiab] OR "1 night of stay"[tiab] OR "one night admission*"[tiab] OR "one night of admission*"[tiab] OR "1 night of admission*"[tiab] OR "1 night of admission*"[tiab] OR "day procedure*"[tiab]) OR ("day car*"[ot] OR daycar*[ot] OR "partial hospitali*"[ot] OR "ambulatory car*"[ot] OR outpatient*[ot] OR "out patient*"[ot] OR "office surg*"[ot] OR "day surg*"[ot] OR "ambulatory surg*"[ot] OR "day of surgery discharg*"[ot] OR "same day discharg*"[ot] OR "ambulant surg*"[ot] OR "ambulant therap*"[ot] OR "ambulant car*"[ot] OR "ambulatory treat*"[ot] OR "ambulant treat*"[ot] OR "outward patient*"[ot] OR policlinic*[ot] OR polyclinic*[ot] OR "ambulatory colectom*"[ot] OR "ambulant colectom*"[ot] OR SDD[ot] OR "same day colectom*"[ot] "Outpatient monitoring"[ot] OR " OR "Ambulatory Monitoring"[ot] OR "Outpatient monitoring"[ot] OR "Monitoring, Outpatient"[ot] OR "Ambulatory Monitoring"[ot] OR "23 hour* of stay"[ot] OR "23 hour* stay" [ot] OR "23 hour* stays"[ot] OR "24 hour* of stay"[ot] OR "24 hour* stay" [ot] OR "24 hour* stays"[ot] OR "1 day* of stay"[ot] OR "1 day* stay" [ot] OR "one day stay*"[ot] OR "one day* stay"[ot] OR "48 hour* of stay"[ot] OR "48 hour* stay" [ot] OR "48 hour* stays"[ot] OR "2 day* of stay"[ot] OR "2 day* stay" [ot] OR "two day* of stay"[ot] OR "two day* stay"[ot] OR "two day* stays"[ot] OR "one night stay*"[ot] OR "one night of stay"[ot] OR "1 night stay"[ot] OR "1 night of stay"[ot] OR "one night admission*"[ot] OR "one night of admission*"[ot] OR "1 night of admission*"[ot] OR "1 night of admission*"[ot] OR "day procedure*"[ot])** Sort by: **Most Recent** |
| #12 | Search: **("Enhanced Recovery After Surgery"[Mesh] OR "Enhanced Recover*”[tiab] OR "Enhanced Recover*"[ot] OR "Enhanced Postsurgical Recover*"[tiab] OR ERAS[tiab] OR "Enhanced Postsurgical Recover*"[ot] OR ERAS[ot] ) AND (accelerat*[tiab] OR enhanced[tiab] OR fast[tiab] OR rapid[tiab] OR "fast track"[tiab] OR "short stay*"[tiab] OR "2.0"[tiab] OR "3.0"[tiab] OR accelerat*[ot] OR enhanced[ot] OR fast[ot] OR rapid[ot] OR "fast track"[ot] OR "short stay*"[ot] OR "2.0"[ot] OR "3.0"[ot]) OR (("Critical Path*"[tiab] OR "Care Map*"[tiab] OR "care path*"[tiab] OR "Clinical Path*"[tiab] OR controlled path*[tiab]) OR "Critical Path*"[ot] OR "Care Map*"[ot] OR "care path*"[ot] OR "Clinical Path*"[ot] OR controlled path*[ot]) AND (accelerat*[tiab] OR enhanced[tiab] OR fast[tiab] OR rapid[tiab] OR "fast track"[tiab] OR "short stay*"[tiab] OR accelerat*[ot] OR enhanced[ot] OR fast[ot] OR rapid[ot] OR "fast track"[ot] OR "short stay*"[ot]))** Sort by: **Most Recent** |
| #11 | Search: **"Critical Pathways"[Mesh]** Sort by: **Most Recent** |
| #10 | Search: **"Day Care, Medical"[Mesh]** Sort by: **Most Recent** |
| #9 | Search: **"Outpatient Clinics, Hospital"[Mesh:NoExp]** Sort by: **Most Recent** |
| #8 | Search: **"Ambulatory Care Facilities"[Mesh]** Sort by: **Most Recent** |
| #7 | Search: **"Outpatients"[Mesh]** Sort by: **Most Recent** |
| #6 | Search: **"Ambulatory Care"[Mesh:NoExp]** Sort by: **Most Recent** |
| #5 | Search: **"Ambulatory Surgical Procedures"[Mesh]** Sort by: **Most Recent** |
| #4 | Search: **"Monitoring, Ambulatory"[Mesh:NoExp]** Sort by: **Most Recent** |

**Embase**

'colon tumor'/de OR 'colon cancer'/exp OR 'colorectal tumor'/exp OR 'large intestine tumor'/de OR 'appendix tumor'/exp OR 'cecum tumor'/exp OR 'large intestine cancer'/exp OR ((carcinoma*:ti,ab,kw OR neoplas*:ti,ab,kw OR tumour*ti,ab,kw OR adenocar*ti,ab,kw OR adenoid* ti,ab,kw OR tumor* ti,ab,kw OR cancer* ti,ab,kw OR malignan* ti,ab,kw) AND ('large intestine'/de OR 'cecum'/exp OR 'colon'/exp OR colorectal*:ti,ab,kw OR colon* ti,ab,kw OR appendi*:ti,ab,kw OR cecum*:ti,ab,kw OR coecum*:ti,ab,kw OR caecum*:ti,ab,kw OR cecal*:ti,ab,kw OR coecal*:ti,ab,kw OR caecal*:ti,ab,kw OR sigmoid*:ti,ab,kw OR CRC:ti,ab,kw))

OR

((laparoscopy/de OR laparoscop*:ti,ab,kw OR ‘minimally invasive surgery’/exp OR ‘mini* invasive surg*’:ti,ab,kw) AND ('large intestine'/de OR 'cecum'/exp OR 'colon'/exp OR colorectal*:ti,ab,kw OR colon* ti,ab,kw OR appendi*:ti,ab,kw OR cecum*:ti,ab,kw OR coecum*:ti,ab,kw OR caecum*:ti,ab,kw OR cecal*:ti,ab,kw OR coecal*:ti,ab,kw OR caecal*:ti,ab,kw OR sigmoid*:ti,ab,kw OR CRC:ti,ab,kw))

OR

('colorectal surgery’/exp) OR 'colon and rectal surg*':ti,ab,kw OR ‘colorectal surg*’:ti,ab,kw OR (‘colectomy'/exp) OR ‘colectom*’:ti,ab,kw OR ‘colon resection*’:ti,ab,kw OR ('large intestine resection'/de) OR ‘large intestin* resection*’:ti,ab,kw OR ‘large bowel resection*’:ti,ab,kw OR ‘hemicolectom*’:ti,a,kw OR ('cecum resection'/exp) OR ‘cecum resection*’:ti,ab,kw OR ‘caec* resection*’:ti,ab,kw OR ‘cecal resection*’:ti,ab,kw OR ‘coecum resection*’:ti,ab,kw OR ‘caecectomy’:ti,ab,kw OR ‘cecectomy’:ti,ab,kw OR ‘coecectomy’:ti,ab,kw OR ('colon surgery'/exp’) OR ‘colon* surg*’:ti,ab,kw

('ambulatory care'/exp OR 'ambulatory surgery'/exp OR 'outpatient department'/exp OR 'outpatient'/exp OR 'day care'/de OR 'adult day care'/exp OR 'same day discharge'/exp OR 'fast track surgery'/exp OR 'fast track protocol'/exp OR 'partial hospitalization'/exp OR 'ambulatory monitoring'/exp)

OR

('ambulatory car*':ti,ab,kw OR 'ambulatory care center*':ti,ab,kw OR 'ambulatory service*':ti,ab,kw OR 'dispensary care':ti,ab,kw OR 'extramural care':ti,ab,kw OR 'office visit*':ti,ab,kw OR 'ambulatory surg*':ti,ab,kw OR 'ambulatory surgical procedur*':ti,ab,kw OR ‘daysurger*’:ti,ab,kw OR ‘outpatient surger*’:ti,ab,kw OR ‘ambulatory surg*’:ti,ab,kw OR 'outpatient department*':ti,ab,kw OR ‘outpatient monitoring’:ti,ab,kw OR ‘office surg*’:ti,ab,kw OR ‘day NEAR/3 surg*’:ti,ab,kw OR ‘ambulant surg*’:ti,ab,kw OR ‘ambulatory surg*’:ti,ab,kw OR ‘ambulatory monitoring’:ti,ab,kw OR ‘ambulant ther*’:ti,ab,kw OR ‘ambulant car*’:ti,ab,kw OR ‘ambulatory treat*’:ti,ab,kw OR ‘ambulant treat*’:ti,ab,kw OR ‘outward patient*’:ti,ab,kw OR ‘ambulatory colectom*’:ti,ab,kw OR ‘ambulant colectom*’:ti,ab,kw

OR 'ambulant operation room*':ti,ab,kw OR 'ambulatory car*':ti,ab,kw OR 'day clinic*':ti,ab,kw OR 'day clinic':ti,ab,kw OR 'day hospital*':ti,ab,kw OR 'out patient*':ti,ab,kw OR ‘outpatient’:ti,ab,kw OR 'outdoor clinic*':ti,ab,kw OR 'policlinic*':ti,ab,kw OR 'polyclinic*':ti,ab,kw OR 'surgicenter*':ti,ab,kw

OR 'day car*’:ti,ab,kw OR 'day care cent*':ti,ab,kw OR 'day cent*':ti,ab,kw OR 'daycare':ti,ab,kw OR 'medical day care':ti,ab,kw OR daycent*:ti,ab,kw

OR 'adult day car*':ti,ab,kw OR 'adult day care cent*':ti,ab,kw OR 'adult day cent*':ti,ab,kw OR 'adult daycare':ti,ab,kw OR 'adult daycenter*':ti,ab,kw

OR 'same day discharge*’:ti,ab,kw OR ‘same day colectom*’;ti,ab,kw OR 'fast track surger*':ti,ab,kw OR 'fast track protocol*':ti,ab,kw OR ‘fast trac*’:ti,ab,kw OR 'partial hospitali*':ti,ab,kw OR ‘rapid recover*’:ti,ab,kw

OR ’23 hour* NEAR/3 stay*’:ti,ab,kw OR ’24 hour* NEAR/3 stay*’:ti,ab,kw OR ‘1 day NEAR/3 stay*’:ti,ab,kw OR ‘one day NEAR/3 stay*’:ti,ab,kw OR ‘48 hour* NEAR/3 stay*’:ti,ab,kw OR ‘2 day* NEAR/3 stay*’:ti,ab,kw OR ‘one night NEAR/3 stay*’:ti,ab,kw OR ‘one night NEAR/3 admission*’:ti,ab,kw OR ‘day procedure*’:ti,ab,kw OR ‘1 night NEAR/3 stay’:ti,ab,kw OR ‘1 night NEAR/3 admission’:ti,ab,kw OR ‘two day* NEAR/3 stay’:ti,ab,kw

OR ‘same day colectom*’:ti,ab,kw OR ‘same day discharg*’:ti,ab,kw OR ‘SDD’:ti,ab,kw)

OR

(('clinical pathway'/exp OR 'enhanced recovery after surgery'/exp) AND (accelerat*:ti,ab,kw OR enhanced:ti,ab,kw OR fast:ti,ab,kw OR rapid:ti,ab,kw OR "fast track":ti,ab,kw OR "short stay*":ti,ab,kw OR 2.0:ti,ab,kw OR 3.0:ti,ab,kw OR

(('clinical pathway*':ti,ab,kw OR 'critical path*':ti,ab,kw OR ‘care path*’:ab,ti,kw OR 'enhanced NEAR/3 recovery*’:ti,ab,kw OR ‘ERAS’:ti,ab,kw OR ‘controlled path*’:ti,ab,kw) AND (accelerat*:ti,ab,kw OR enhanced:ti,ab,kw OR fast:ti,ab,kw OR rapid:ti,ab,kw OR "fast track":ti,ab,kw OR "short stay*":ti,ab,kw))

**Web of Science**

1: TS=(("Colorectal Neoplasms" OR "Appendiceal Neoplasms"))

2: TS=(((carcinoma* OR neoplas* OR tumour* OR adenocar* OR adenoid* OR tumor* OR cancer* OR malignan*) AND ("Intestine, Large” OR Cecum OR Colon OR colorectal* OR colon* OR appendi* OR cecum* OR coecum* OR caecum* OR cecal* OR coecal* OR caecal* OR sigmoid* OR CRC)))

3: TS=("Colorectal Surgery" OR "Colon and Rectal Surg* " OR "Colorectal Surg*" OR "Colon Surg* " OR "Colectomy" OR "Colectom*" OR "Large Bowel Resection*" OR "Hemicolectom*" OR "Colon surg*" OR "Colonic surg*" OR "Colorectal resection*" OR "Colon resection*")

4: TS=(("Enhanced Recover*” OR "Enhanced Postsurgical Recover*" OR ERAS OR (accelerat* OR enhanced OR fast OR rapid OR "fast track" OR "short stay*" OR "2.0" OR "3.0")) AND ("Critical Path*" OR "Care Map*" OR "care path*" OR "Clinical Path*" OR controlled path*))

5: TS=("day car*" OR daycar* OR "partial hospitali*" OR "ambulatory car*" OR outpatient* OR "out patient*" OR "office surg*" OR "day surg*" OR "ambulatory surg*" OR "day of surgery discharg*" OR "same day discharg*" OR "ambulant surg*" OR "ambulant therap*" OR "ambulant car*" OR "ambulatory treat*" OR "ambulant treat*” OR "outward patient*" OR policlinic* OR polyclinic* OR "ambulatory colectom*" OR "ambulant colectom*" OR SDD OR "same day colectom*" OR "Outpatient monitoring" OR "Ambulatory Monitoring" OR "23 hour* of stay" OR "23 hour* stay" OR "23 hour* stays" OR "24 hour* of stay" OR "24 hour* stay" OR "24 hour* stays" OR "1 day* of stay" OR "1 day* stay" OR "one day* of stay" OR "one day stay*" OR "48 hour* of stay" OR "48 hour* stay" OR "48 hour* stays" OR "2 day* of stay" OR "2 day* stay" OR "two day* of stay" OR "two day* stay" OR "two day* stays" OR "one night stay*" OR "one night of stay" OR "1 night stay" OR "1 night of stay" OR "one night admission*" OR "one night of admission*" OR "1 night of admission*" OR "1 night of admission*" OR "day procedure*")

6: TS=(((“clinical pathway*” OR “critical path*” OR “pathway, clinical” OR “care map*” OR “controlled path*” OR “care path*”) AND (enhanced OR accelerat* OR fast OR rapid OR “fast track” OR “short stay*" OR 2.0 OR 3.0)))

**Cochrane library**

1 MeSH descriptor: [Colorectal Neoplasms] explode all trees

#2 MeSH descriptor: [Appendiceal Neoplasms] explode all trees

#3 MeSH descriptor: [Intestine, Large] this term only

#4 MeSH descriptor: [Cecum] explode all trees

#5 MeSH descriptor: [Colon] explode all trees

#6 carcinoma* OR neoplas* OR tumour* OR adenocar* OR adenoid* OR tumor* OR cancer* OR malignan*:ti,ab,kw

#7 #3 OR #4 OR #5

#8 colorectal* OR colon* OR appendi* OR cecum* OR coecum* OR caecum* OR cecal* OR coecal* OR caecal* OR sigmoid* OR CRC:ti,ab,kw

#9 ((#7 OR #8) AND #6) OR #1 OR #2

#10 MeSH descriptor: [Monitoring, Ambulatory] this term only

#11 MeSH descriptor: [Ambulatory Surgical Procedures] explode all trees

#12 MeSH descriptor: [Outpatient Clinics, Hospital] this term only

#13 MeSH descriptor: [Day Care, Medical] explode all trees

#14 "day car*" OR daycar* OR "partial hospitali*" OR ("ambulatory 3n car*" ) OR outpatient* OR "out patient*" OR "office surg*" OR "day surg*" OR "ambulatory surg*" OR ("day n5 of surgery discharg*") OR "same day discharg*" OR ("ambulant 3n surg*") OR ("ambulant 3n therap*") OR ("ambulant 3n car*") OR ("ambulatory 3n treat*") OR ("ambulant 3n treat*") OR "outward patient*" OR policlinic* OR polyclinic* OR ("ambulatory 3n colectom*") OR ("ambulant 3n colectom*") OR SDD OR "same day colectom*" OR "outpatient monitoring" OR "Ambulatory Monitoring" OR "23 hour stay*" OR "24 hour stay*" OR "1 day stay*" OR "48 hour stay*" OR "2 day stay*" OR "one night stay*" OR "one night admission*" OR "day procedure*":ti,ab,kw

#15 MeSH descriptor: [Ambulatory Care] this term only

#16 MeSH descriptor: [Outpatients] explode all trees

#17 MeSH descriptor: [Ambulatory Care Facilities] explode all trees

#18 #10 OR #11 OR #12 OR #13 OR #15 OR #16 OR #17 OR #14

#19 MeSH descriptor: [Critical Pathways] explode all trees

#20 "Critical Path*" OR "Care Map*" OR "care path*" OR "Clinical Path*":ti,ab,kw

#21 #19 OR #20

#22 accelerat* OR fast OR rapid OR "fast track" OR "short stay*":ti,ab,kw

#23 #21 AND #22

#24 #18 OR #23

#25 #9 AND #24

#26 MeSH descriptor: [Laparoscopy] this term only

#27 laparoscop*:ti,ab,kw

#28 MeSH descriptor: [Minimally Invasive Surgical Procedures] this term only

#29 ("minimally invasive surgery"):ti,ab,kw (Word variations have been searched)

#30 colorectal* OR colon* OR appendi* OR cecum* OR coecum* OR caecum* OR cecal* OR coecal* OR caecal* OR sigmoid* OR CRC:ti,ab,kw

#31 MeSH descriptor: [Intestine, Large] this term only

#32 MeSH descriptor: [Colon] explode all trees

#33 MeSH descriptor: [Cecum] explode all trees

#34 "day car*" OR daycar* OR "partial hospitali*" OR ("ambulatory 3n car*" ) OR outpatient* OR "out patient*" OR "office surg*" OR "day surg*" OR "ambulatory surg*" OR ("day n5 of surgery discharg*") OR "same day discharg*" OR ("ambulant 3n surg*") OR ("ambulant 3n therap*") OR ("ambulant 3n car*") OR ("ambulatory 3n treat*") OR ("ambulant 3n treat*") OR "outward patient*" OR policlinic* OR polyclinic* OR ("ambulatory 3n colectom*") OR ("ambulant 3n colectom*") OR SDD OR "same day colectom*" OR "outpatient monitoring" OR "Ambulatory Monitoring" OR "23 hour stay*" OR "24 hour stay*" OR "1 day stay*" OR "48 hour stay*" OR "2 day stay*" OR "one night stay*" OR "one night admission*" OR "day procedure*"ti,ab,kw (Word variations have been searched)

#35 enhanced OR accelerat* OR fast OR rapid OR "fast track" OR "short stay*" OR 2.0 OR 3.0:ti,ab,kw

#36 "Critical Path*" OR "Care Map*" OR "care path*" OR "Clinical Path*":ti,ab,kw

#37 MeSH descriptor: [Colorectal Surgery] explode all trees

#38 MeSH descriptor: [Colectomy] this term only

#39 ("Colon and Rectal Surg*") OR ("Colorectal Surg*") OR ("Colon Surg*") OR ("Colon and Rectal Surg*") OR ("Colectom*") OR ("Large Bowel Resection*") OR ("Hemicolectom*"):ti,ab,kw (Word variations have been searched)

#40 #39 OR #38 OR #37 OR ((#33 OR #32 OR #31 OR #30) AND (#29 OR #28 OR #27 OR #26))

#41 #10 OR #11 OR #12 OR #13 OR #15 OR #16 OR #17 OR #34

#42 #41 OR (#21 AND #35)

#43 #9 OR #40

#44 #43 AND #42

#45 #44 NOT #25
